# An Enhanced Method to Estimate Heart Rate from Seismocardiography via Ensemble Averaging of Body Movements at Six Degrees of Freedom

**DOI:** 10.3390/s18010238

**Published:** 2018-01-15

**Authors:** Hyunwoo Lee, Hana Lee, Mincheol Whang

**Affiliations:** 1 Department of Emotion Engineering, University of Sangmyung, Seoul 03016, Korea; lhw4846@naver.com (H.L.); hlee1844@gmail.com (Ha.L.); 2 Department of Intelligence Informatics Engineering, University of Sangmyung, Seoul 03016, Korea

**Keywords:** accelerometer, gyroscope, heart rate measurement, seismocardiography (SCG), wearable device, ensemble averaging

## Abstract

Continuous cardiac monitoring has been developed to evaluate cardiac activity outside of clinical environments due to the advancement of novel instruments. Seismocardiography (SCG) is one of the vital components that could develop such a monitoring system. Although SCG has been presented with a lower accuracy, this novel cardiac indicator has been steadily proposed over traditional methods such as electrocardiography (ECG). Thus, it is necessary to develop an enhanced method by combining the significant cardiac indicators. In this study, the six-axis signals of accelerometer and gyroscope were measured and integrated by the L2 normalization and multi-dimensional kineticardiography (MKCG) approaches, respectively. The waveforms of accelerometer and gyroscope were standardized and combined via ensemble averaging, and the heart rate was calculated from the dominant frequency. Thirty participants (15 females) were asked to stand or sit in relaxed and aroused conditions. Their SCG was measured during the task. As a result, proposed method showed higher accuracy than traditional SCG methods in all measurement conditions. The three main contributions are as follows: (1) the ensemble averaging enhanced heart rate estimation with the benefits of the six-axis signals; (2) the proposed method was compared with the previous SCG method that employs fewer-axis; and (3) the method was tested in various measurement conditions for a more practical application.

## 1. Introduction

Continuous cardiac monitoring is necessary for the diagnosis of cardiovascular disease. A medical emergency caused by a cardiac arrest can be prevented by continuous cardiac monitoring especially from wearable devices. In recent years, the advancement of novel instruments has allowed the monitoring of cardiovascular activity outside of clinical environments. One of the vital components to develop the monitoring system is seismocardiography (SCG). SCG is non-invasively measured from the thoracic movements produced by the contraction of heart and the ejection of the blood from ventricles into the vasculature [1] using an accelerometer sensor. SCG makes cardiac monitoring more comfortable than traditional methods, such as electrocardiography (ECG) or photoplethysmography (PPG). Recently, SCG has drawn interest due to the development of microelectromechanical systems (MEMS) technology.

Initial studies on SCG only focused on the z-axis accelerometer signal [1,2]. They considered the z-axis accelerometer signal important because it measures dorso-ventral direction motion induced by the heartbeat on the chest. Since instrumentation and signal processing technology have advanced, the x-axis and y-axis accelerometer signals have also been employed to monitor the cardiac activity. Additional cardiac information could be derived from the analysis of the tri-axis accelerometer signals [3,4,5] and their vector trajectory [6] during the heart cycle. Pandia et al. [5] showed that the y-axis of accelerometer can be a reference signal to cancel the motion artifacts. Paukkunen et al. [6] suggested that the spatial distribution of tri-axis accelerometer signals may be useful to classify healthy individuals and those with heart disease. The cardiac cycle events based on annotation of SCG (e.g., MC, AO, AC, and MO) were quantified by the distribution and distance in octants. Migeotte et al. [4] proposed an integrated method for analyzing tri-axis accelerometer signals as the kinetic energy and showed the signals were significant parameters for physiological interpretation of SCG.

Recent SCG studies have investigated the gyroscope signal to enhance the cardiac monitoring. Tadi et al. [7] proposed gyrocardiography (GCG) to monitor the cardiac activity from three-dimensional angular velocity signals measured on the chest using a gyroscope sensor. They demonstrated that the GCG can provide information of the tissue velocity and relative strain rate of the myocardium. Migeotte et al. [8] proposed the Multi-dimensional Kineticardiography (MKCG) to integrate tri-axis gyroscope signals as the kinetic energy. They showed that the rotational components of MKCG are particularly important for the cardiac cycle. Yang et al. [9] developed an automatic annotation of SCG using the gyroscope. They integrated the tri-axis gyroscope signals as the kinetic energy with the MKCG approach [8] and proved that their peaks are associated with the annotation of SCG. Jia et al. [10] showed that the heartbeat-induced peaks could be significantly observed in the waveform of the gyroscope rather than the accelerometer, so they focused on the analysis of the gyroscope signal and estimated the heart rate from a chest-worn gyroscope sensor.

Despite the steadily proposed methods of the novel cardiac indicators, SCG has presented lower accuracy compared to traditional methods (e.g., ECG). It is necessary to develop an enhanced method by combining the significant cardiac indicators. The statistical methods have been applied to reduce noise and amplify deterministic signals from random signals. The ensemble averaging for repeatedly measured accelerometer signals have been demonstrated to reduce noise on SCG [5], which allowed for the application of SCG on cardiac monitoring in daily life. The L2 normalization for different components of the motion signal has been proven to make the ballistocardiogram (BCG) more robust to different body postures [11]. For reference, the BCG is to monitor the cardiac activity from movement measured on other body locations (e.g., head, arms, or foots) besides the chest [12]. The MKCG [8] integrated the tri-axis accelerometer signals and tri-axis gyroscope signals as linear and rotational kinetic energy, respectively. They showed that the rotational components are more important than the linear components for a signal cardiac cycle. Although they integrated linear and rotational components as total kinetic energy, it was only used to compare the importance of linear and rotational components, without the benefit of their integration.

Consequently, recent studies on SCG have been aimed for more distinct measurements by combining the additional information of motion relevant to heartbeat. However, they tried to combine multi-axis of the same sensor not other sensors, even though the peaks of the acceleration and angular velocity of thoracic movements had associated each other. Thus, this study hypothesized that the accuracy of SCG can be increased by combining the accelerometer and gyroscope signals, which is the significant cardiac indicators represented acceleration and angular velocity of the thoracic movements derived from heartbeat.

This study was conducted to develop a novel method to combine the six-axis accelerometer and gyroscope signals and to enhance practical application of the cardiac monitoring. The focus was on the heart rate estimation to demonstrate the proposed method, since it is the most popular indicator to monitor the cardiac activity. The signals are sensitive to physical conditions and postures. Therefore, they were experimented to estimate the heart rate in the relaxed and aroused conditions during standing and sitting postures. The ECG was simultaneously measured to verify the accuracy of heart rate estimation from SCG. The proposed method combined the six-axis accelerometer and gyroscope signals. It was compared with the traditional SCG method, which only employed the z-axis or tri-axis accelerometer signal. The contributions of this study can be summarized as follows: (1) the ensemble averaging enhanced heart rate estimation with the benefits of the six-axis signals; (2) the proposed method was compared with the previous SCG method that employs fewer-axis; and (3) the method was tested in various measurement conditions for a more practical application.

## 2. Methods

### 2.1. Participants

A power analysis was conducted to determine the required number of samples for valid inference in this study. Two-independent Pearson’s correlation was used to assess the hypothesis. The power analysis was conducted using G * Power [13]. In order to achieve 0.80 statistical power at α = 0.05 (two tailed) using an Pearson’s correlation, the effect size was estimated as 0.80 and the number of samples needed be at least 28 participants in each group. Thus, thirty participants (15 males) who were at an average age of 27.3 ± 3.3 participated in the experiment. They were paid with an incentive and had no medical history related to cardiovascular diseases. All participants were asked to get enough sleep and abstain from alcohol, caffeine, and cigarettes before the experiment. Written informed consent was obtained from all participants prior to the experiment. The study population incorporated only the healthy and young individuals, so that it is limited to provide definitive and conclusive results. It is necessary to verify our method for older individuals and cardiac patients in the future.

### 2.2. Apparatus

**Figure 1:**
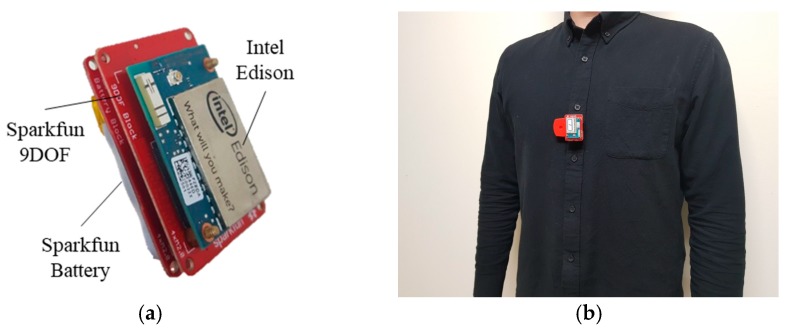
Overview of the apparatus. (**a**) Packaging of the Intel Edison, Sparkfun 9DOF, and Sparkfun Battery; (**b**) Appearance of the apparatus when worn by participant.

This study measured SCG from the chest utilizing a portable and wearable sensor, which has the MEMS accelerometer and gyroscope. The apparatus was comprised of three modules such as an Intel Edison, a Sparkfun 9DOF, and a Sparkfun Battery (see Figure 1). Intel Edison is a microcomputer with a built-in Bluetooth and Wi-Fi function, a 1 GB of DDR3 RAM, and 4GB of flash storage. Sparkfun 9DOF is a MEMS motion sensor, which includes a tri-axis accelerometer and a tri-axis gyroscope. Intel Edison has power consumption of 350 mW (3.3 V/100 mA) and is powered through the Sparkfun battery, which has a single cell Lipo Charger and 400 mAh battery. Intel Edison is also connected to other modules via the I2C serial bus interface. The apparatus was 45 × 30 × 20 mm big that weighed 30 g so that the subjects could simply clip it on their clothes. The *x*-axis is the left-to-right direction, *y*-axis is the foot-to-head direction, and *z*-axis is the dorso-to-ventral direction.

### 2.3. Experimental Procedure

**Figure 2:**
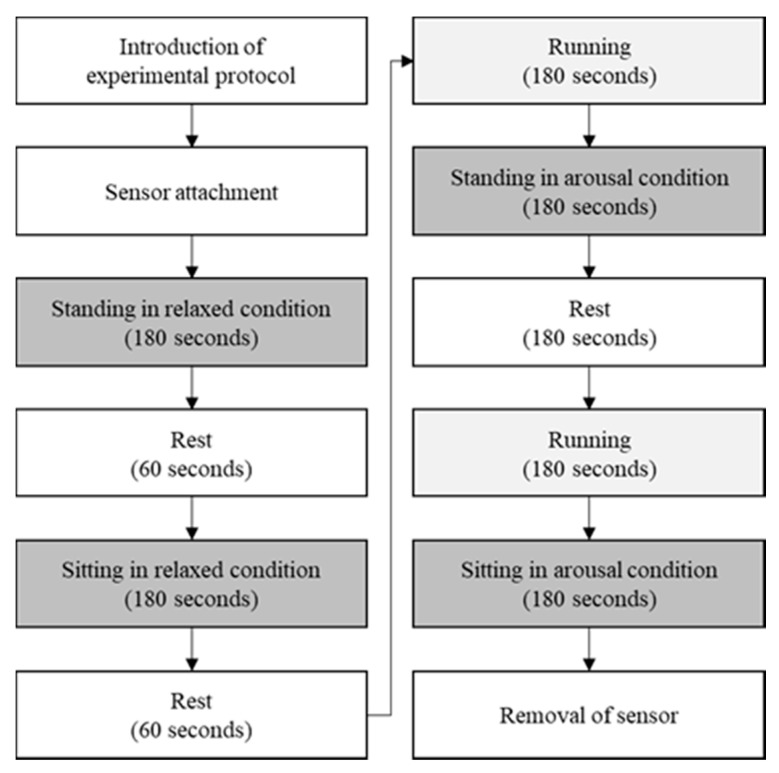
Experimental procedure according to two physical conditions and two body postures. The experiment was proceeded for a total of 30 min, and the sufficient rest was given between each task.

One of the challenges for SCG is that the accelerometer signal is sensitive to body postures. To ensure clinical relevance, it is necessary to test SCG for a wide range of heart rates. Therefore, this study experimented the proposed method in various physical conditions and postures, as shown in Figure 2.

SCG was measured when participants were physically relaxed or aroused during standing or sitting postures. To ensure relaxation, participants were asked to close their eyes and maintain body posture for 3 min. For arousal, participants were asked to maintain body postures for 3 min after running at a speed of 8.5 km/h on a treadmill for 3 min. These procedures were repeated twice during two different body postures (i.e., standing and sitting). The experimental procedure was 30 min long and sufficient rest was given between each tasks. Longer rest time was given after arousal tasks due to the time for cardiac activity to be restored back to steady condition. This protocol was approved by the Institutional Review Board of the Sangmyung University, Seoul, Korea (BE2016-14).

The accelerometer and gyroscope signals were measured with the SCG measurement device developed in this study, and the ECG was measured by the Lead-I simultaneously. The ECG served as a ground-truth for the evaluation of SCG. The ECG was measured at sampling rate of 512 Hz by a system consisting of a ECG 100C amplifier system and a MP150 data acquisition system (BIOPAC Systems Inc., Goleta, CA, USA). Participants were asked to minimize the movement of their arms to reduce the noise while measuring the ECG.

### 2.4. Data Acquisition and Processing

The accelerometer and gyroscope signals were recorded to a local flash storage in our apparatus for minimizing data loss, even though they can be measured by connecting to another Bluetooth device in real time. In addition, the system for data acquisition has been operated with the multi-threading for real-time data processing in the future. After the experiment, the signals were analyzed on a desktop with Python. SCG was determined by analyzing and combining both the accelerometer and gyroscope signals as described below.

**Figure 3:**
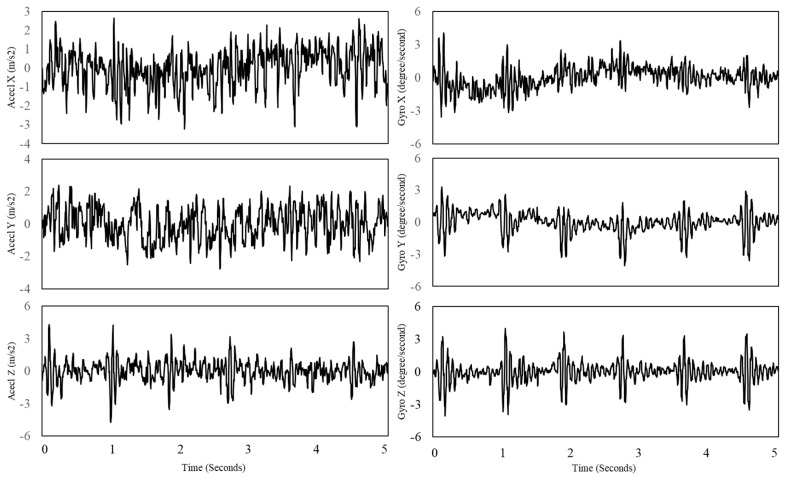
Raw signals of tri-axis of accelerometer (**Left**) and tri-axis of gyroscope (**Right**).

First, the raw signals were measured with the sampling rates within the ranges of around 100 and 200 Hz. The sampling rates for the accelerometer and gyroscope are slightly different due to the data acquisition system based on multi-threading. Their sampling rates were fixed at 256 Hz to match the length of the signals for their integration. This study employed the cubic spline interpolation [14], which estimated cubic polynomials between each of the data points as the acceleration and angular velocity has non-linear property. The raw signals of the accelerometer and gyroscope are depicted in Figure 3.

**Figure 4:**
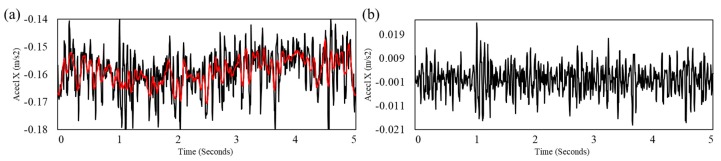
Example of motion cancellation by the Savitzky-Golay filter with order of 2 and window size of 31. (**a**) Raw signal (black) and estimated noise (red); (**b**) Corrected signal.

Second, the motion-induced noise was estimated by the Savitzky-Golay filter (order = 2, window size = 31 samples) [15]. Because this step is to correct the baseline of the disrupted signal due to the motion-induced noise, the window size of the filter was empirically chosen to best estimate the baseline of the signal as shown in Figure 4. If the signal consists of a set $n\left\{x_{i},\;y_{i}\right\}$ with points (i=1, …, n), the noise N was calculated as:

(1) $$N_{i}=savgol\left(y\right)=\sum_{j=-\frac{m-1}{2}}^{\frac{m-1}{2}}C_{j}y_{i+j},$$

where x is an independent variable, $y_{i}$ is an observed value, savgol is a function of the Savitzky-Golay filter, $C_{j}$ is a set of m convolution coefficients of the Savitzky-Golay filter, and m is a window size of the filter (i.e., m=31). The corrected signal $\bar{y}$ was calculated as:

(2) $$\bar{y}=y-N$$

**Figure 5:**
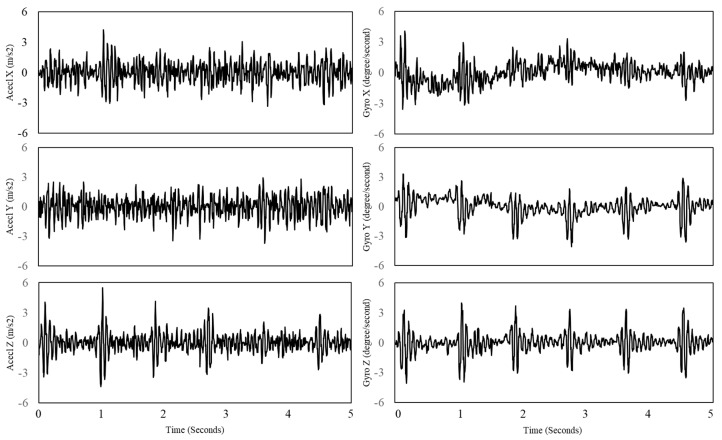
Corrected signals of tri-axis of accelerometer (**Left**) and tri-axis of gyroscope (**Right**) by the Savitzky-Golay filter.

The noise was subtracted from each signal to reduce the motion artifacts. The Savitzky-Golay filter is able to preserve higher order moments around inflection points [5], thus, it maximizes the cancellation of the noise induced by motion and maximize loss of information induced by heartbeat. The corrected signals of the accelerometer and gyroscope are depicted in Figure 5.

Third, the kinetic energy waveforms of accelerometer and gyroscope were calculated from tri-axis signals of accelerometer and gyroscope by L2-normalization and multi-dimensional kineticardiography (MKCG) approaches [8], respectively:

(3) $$E_{accel}=\sqrt{v_{x}{}^{2}+v_{y}{}^{2}+v_{z}{}^{2}}$$

where $E_{accel}$ is a energy waveform of accelerometer, and v is a linear velocity of the sensor.

(4) $$E_{gyro}=\frac{1}{2}\left(I_{x}w_{x}{}^{2}+I_{y}w_{y}{}^{2}+I_{z}w_{z}{}^{2}\right)$$

where $E_{gyro}$ is a kinetic energy waveform of gyroscope, I is moments of inertia of the sensing unit, and w is an angular speed. The moments of inertia were estimated by linear regression algorithm proposed by [9]. Then, the 2nd order Butterworth bandpass filters with the cut-off frequencies of 0.8–10 Hz and 1–20 Hz were applied on the kinetic energy waveforms of accelerometer and gyroscope, respectively. Their pass bands were determined by SCG (0.8–10 Hz) and GCG (1–20 Hz) approaches [2,7], and their order were determined as the order of 2, because the filters with higher order lead to a significant biophysical information loss in the waveform.

**Figure 6:**
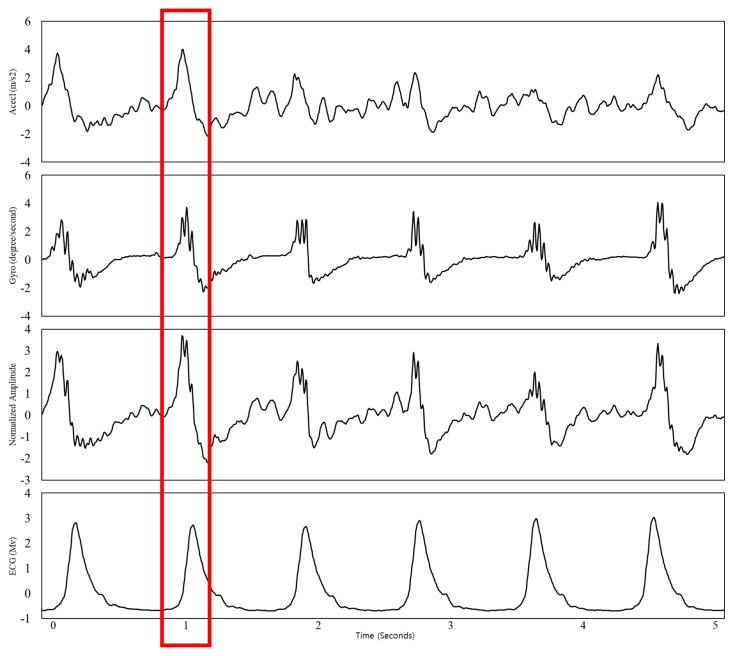
Waveforms of the accelerometer, gyroscope, their ensemble averaging, and synchronized ECG. The red box indicates how each peak of the waveforms corresponds to the peak of ECG.

Forth, SCG was finally determined by the normalization and ensemble averaging of the energy waveforms of the accelerometer and gyroscope. The waveforms were standardized by the z-score normalization and combined via the ensemble averaging:

(4) $$E_{ea}=\frac{\left(E_{accel}+E_{gyro}\right)}{2}$$

where $E_{ea}$ is a waveform of ensemble averaging of accelerometer and gyroscope, $E_{accel}$ is a waveform of accelerometer, and $E_{gyro}$ is waveform of gyroscope. This method reduces noise and amplifies the diverse information of the cardiac activity by giving the same relevance to each of the axis, thus, it makes SCG more robust to different body postures. Figure 6 presents the waveforms of the accelerometer, gyroscope, their ensemble averaging, and synchronized ECG.

### 2.5. Heart Rate Estimation

In SCG studies, it was important to select appropriate peak detection algorithms according to the shape of the signals for heart rate estimation. Depending on the type of axis, the peak detection algorithms showed different performances [16]. In this study, the heart rate was calculated by analyzing in frequency domain that was not sensitive to the shape of the signal. Therefore, SCG was transformed to the frequency domain by a Sparse Fast Fourier Transform (SFFT) with a window size of 5 s and an interval size of 1 s. The dominant frequency was identified with the highest power between the ranges of 0.75 and 2.5 Hz. The dominant frequency is related to the Peak-to-Peak Intervals (PPI) since the frequency shows the number of occurrences of repeating peaks. The heart rate of SCG was finally estimated by multiplying by the dominant frequency and 60.

On the other hand, the ECG was clearly measured without noise because the participants were asked to minimize the movement of their arms in experiments. Thus, the heart rate of the ECG was calculated by the QRS detection algorithm, which was implemented by Pan and Tompkins to detect the R peaks [17]. The QRS detection algorithm is more accurately calculate the heart rate from the ECG than the other methods, because it is designed by considering of the shape of the QRS complex.

### 2.6. Statistics

The heart rate with SCG was evaluated by comparing it to the heart rate with the ECG, which was based on their mean absolute error (MAE), standard deviation of absolute error (SDAE), and root mean squared error (RMSE), Pearson’s correlation coefficients (CC), and Bland-Altman plot. The MAE, SDAE, and RMSE were utilized as quantities for evaluating the similarity between predictions and observations. If the calculated error was approaching to the 0, it was indicated as the observations were similar with the predictions. The Pearson’s correlation coefficient was utilized to determine the statistical relationship between the two groups. The correlation coefficient approaching to the 1 was indicated as a strong positive correlation, whereas the value approaching to the −1 indicated as a strong negative correlation. The correlation was indicated as statistically significant if *p*-value was less than 0.05. The Bland-Altman plot was statistic method that was used for the comparison between values which were achieved by two measurement techniques. This method was consistent with the analysis, which combined both the graphical and statistical interpretation. The plot was represented by assigning the mean (x-axis) and difference (y-axis) between the two measurements. The 95% limits of an agreement were calculated by mean difference and the ±1.96 standard deviation of the difference was represented as lines on the plot.

## 3. Results

### 3.1. Estimation of Heart Rate in Relaxed Condition

The heart rates measured from z-axis of accelerometer, tri-axis of accelerometer, and six-axis of accelerometer and gyroscope were evaluated with respected analyses. Table 1 shows the estimated heart rate during standing and sitting postures in relaxed condition. The errors during standing posture were lowest when the six-axis was analyzed (MAE = 2.56, SDAE = 3.69, RMSE = 4.37, CC = 0.948). Similarly, the errors of the estimated heart rate during sitting posture were lowest when the six-axis was considered for the analysis (MAE = 5.25, SDAE = 6.68, RMSE = 8.41, CC = 0.725).

**Figure 7:**
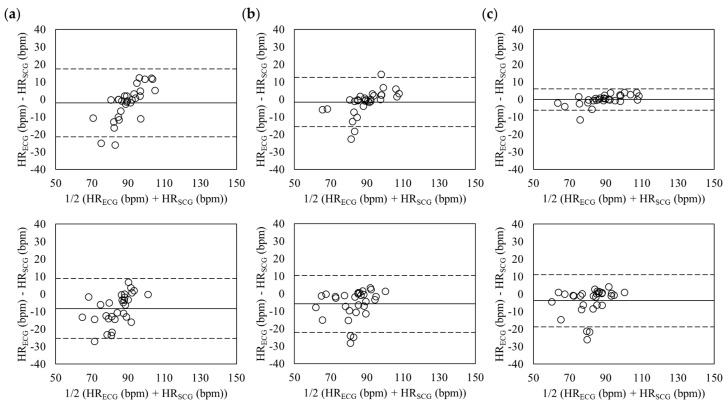
Bland-Altman plots of heart rates estimated from SCG and ECG in relaxed condition during standing (**Top**) and sitting (**Down**) postures based on the z-axis (**a**); tri-axis (**b**); and six-axis (**c**). The lines are the mean errors and 95% LOA.

The Bland-Altman plots of estimated heart rate during standing and sitting postures in relaxed condition are shown in Figure 7. The standing posture, top plots, had the mean errors of −2.02 with 95% limits of agreement (LOA) in −21.45 to 17.41 (z-axis), −1.54 with 95% LOA in −15.65 to 12.57 (tri-axis), and −0.07 with 95% LOA in −6.14 to 6.00 (six-axis). The mean errors during sitting posture (down plots) were −8.30 with 95% LOA in −25.35 to 8.75 (z-axis), −5.86 with 95% LOA in −22.19 to 10.46 (tri-axis), and −3.82 with 95% LOA in −18.70 to 11.07 (six-axis). The differences of the heart rates were lower in the six-axis than the ones in z-axis and tri-axis, which showed that the heart rates were almost synchronized.

### 3.2. Estimation of Heart Rate in Aroused Condition

The heart rates during standing and sitting postures in aroused condition were evaluated as shown in Table 2. The errors during sitting posture were lowest when the six-axis was considered (MAE = 2.33, SDAE = 2.53, RMSE = 3.41, CC = 0.988). The errors during standing posture were also lower in the six-axis than in the other axis (MAE = 2.58, SDAE = 5.69, RMSE = 6.16, CC = 0.940).

**Figure 8:**
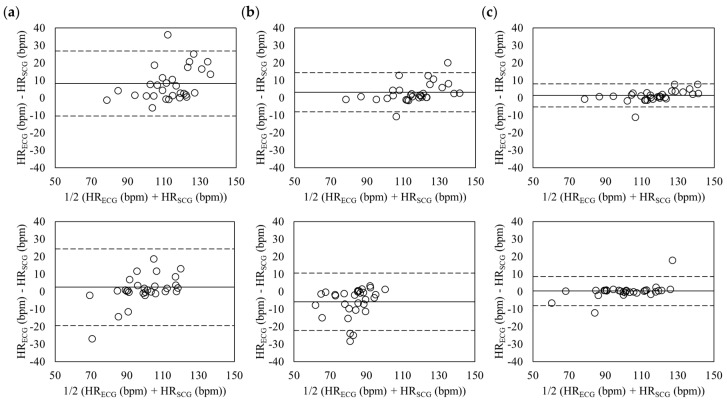
Bland-Altman plots of heart rates estimated from SCG and ECG in aroused condition during standing (**Top**) and sitting (**Down**) postures based on the *z*-axis (**a**); tri-axis (**b**); and six-axis (**c**). The lines are the mean errors and 95% LOA.

The Figure 8 shows the Bland-Altman plots of the estimated heart rate in aroused condition during standing and sitting postures. The mean errors during standing posture (top plots) were 8.14 with 95% LOA in −10.48 to 26.76 (z-axis), 3.17 with 95% LOA in −7.97 to 14.31 (tri-axis), and 1.34 with 95% LOA in −5.19 to 7.87 (six-axis). The sitting posture (down plots) had the mean errors of 2.46 with 95% LOA in −19.50 to 24.42 (z-axis), 0.08 with 95% LOA in −12.23 to 12.38 (tri-axis), and 0.23 with 95% LOA in −8.16 to 8.62 (six-axis). Six-axis during standing and sitting postures showed the lowest differences in the heart rates.

## 4. Discussion

In this study, the heart rate was estimated from SCG of the z-axis, tri-axis, and six-axis in the relaxed and aroused conditions during standing and sitting postures. This study evaluated the accuracy of the proposed method, which combines the six-axis accelerometer and gyroscope signals, and compared with the traditional method which only employ the z-axis or tri-axis accelerometer signal. The performance of our method was much higher than the traditional method in all physical conditions and body postures.

Overall, this research has drawn four significant findings. First, the heart rate from only the accelerometer was more accurately detected when analyzing multiple axes (i.e., tri-axis) than when analyzing the single axis (i.e., z-axis). This result shows that the x- and y- axis of accelerometer also reflect the thoracic movement induced by heartbeat as much as z-axis of one. Although this result has been hypothesized in previous study [16], the performances of the two methods have not been directly compared. Thus, this study strongly encourages the additional axis of the accelerometer as a requirement to estimate the heart rate from SCG more accurately

Second, the heart rate in aroused condition was detected more accurately than in relaxed condition during both standing and sitting postures. This can be interpreted that the accelerometer and gyroscope signals could reflect strong heartbeat better than weak heartbeat due to the large movement of the chest. Thus, it is necessary to analyze the characteristics of the accelerometer and gyroscope signals depending on the physical conditions. If the signal processing was adaptively selected based on the characteristics, the estimation of heart rate may be enhanced. Recent studies by Migeotte et al. [4], Castiglioni et al. [18], and Paukkunen et al. [6] reported significant parameters to interpret physiological characteristics by analyzing spatial distribution of the tri-axis accelerometer signal. These parameters might serve as indicators to select the axis of signals according to the physical conditions.

Third, this study observed significant results for integration of the six-axis. Particularly, their correlation coefficients were higher than results of z-axis and tri-axis. This result indicated that with the advancement of the signal processing for the six-axis, the ECG could eventually be replaced by SCG. Future studies on SCG should consider the use of additional motion sensors, which include the gyroscope.

Finally, this study presented the combination of the accelerometer and gyroscope to improve the accuracy of heart rate estimation from SCG. Despite the low accuracy of our results, a new apparatus was developed for the convenience of a more practical use of SCG in daily life. Previously, wearable SCG measurement systems have been tightly worn on the chest especially on the chest area, near the clavicle [3,18,19], but the proposed apparatus can simply be worn by clipping it on the clothes. This measurement system should be developed and tested to minimize the measurement burden and to develop a wearer-comfortably design of wearable devices.

## 5. Conclusions

This study developed a novel method to estimate the heart rate from SCG by ensemble averaging of the six-axis of accelerometer and gyroscope. Proposed method estimated the heart rate more accurately than the traditional SCG methods in relaxed and aroused conditions during standing and sitting postures. Former SCG measurement methods have used accelerometer and gyroscopes, but they did not focus on combining the two to improve the estimation accuracy. This study implied that a reliable measurement method for integrating multi-signals should be established. The proposed method is expected to increase estimation accuracy of the heart rate, and therefore it takes further steps toward the development of SCG.

## Figures and Tables

**Table 1:** Estimation of heart rate in relaxed condition.

Posture	Signal	MAE	SDAE	RMSE	CC
Standing	Accel *z*	6.97	6.83	9.68	0.658 **
	Accel *xyz*	6.24	5.00	7.94	0.810 **
	Accel + Gyro	**2.56**	**3.69**	**4.37**	0.948 **
Sitting	Accel *z*	8.40	7.20	10.98	0.588 **
	Accel *xyz*	6.77	6.59	9.38	0.633 **
	Accel + Gyro	**5.25**	**6.68**	**8.41**	0.725 **

^1^ Accel *z* = *z*-axis of accelerometer; Accel *xyz* = tri-axis of accelerometer; Accel + Gyro = six-axis of accelerometer and gyroscope; MAE = mean absolute error; SDAE = standard deviation of absolute error; RMSE = root mean square error; CC = Pearson’s correlation coefficient. Two asterisks represent significant correlation levels at *p* < 0.01, respectively. The lowest error and highest correlation values are bolded.

**Table 2:** Estimation of heart rate in aroused condition.

Posture	Signal	MAE	SDAE	RMSE	CC
Standing	Accel *z*	6.91	8.80	11.07	0.794 **
	Accel *xyz*	4.18	4.96	6.43	0.937 **
	Accel + Gyro	**2.33**	**2.53**	**3.41**	0.988 **
Sitting	Accel *z*	5.62	9.50	10.90	0.759 **
	Accel *xyz*	4.35	7.43	8.51	0.877 **
	Accel + Gyro	**2.58**	**5.69**	**6.16**	0.940 **

^1^ Accel *z* = *z*-axis of accelerometer; Accel *xyz* = tri-axis of accelerometer; Accel + Gyro = six-axis of accelerometer and gyroscope; MAE = mean absolute error; SDAE = standard deviation of absolute error; RMSE = root mean square error; CC = Pearson’s correlation coefficient. Two asterisks represent significant correlation levels at *p* < 0.01, respectively. The lowest error and highest correlation values are bolded.
